# Automatic adaptive emotion regulation is associated with lower emotion-related activation in the frontoparietal cortex and other cortical regions with multi-componential organization

**DOI:** 10.3389/fnbeh.2023.1059158

**Published:** 2023-03-06

**Authors:** Motoaki Sugiura, Yoko Katayori, Tomohiko Muratsubaki, Miyuki Shiratori, Sugiko Hanawa, Keyvan Kashkouli Nejad, Daisaku Tamura, Ryuta Kawashima, Shin Fukudo

**Affiliations:** ^1^Institute of Development, Aging and Cancer, Tohoku University, Sendai, Japan; ^2^International Research Institute of Disaster Science, Tohoku University, Sendai, Japan; ^3^Department of Behavioral Medicine, Tohoku University Graduate School of Medicine, Sendai, Japan

**Keywords:** emotion regulation, reappraisal, acceptance, mindfulness, fMRI, prefrontal cortex, spontaneous, implicit

## Abstract

Although some researchers consider automatic adaptive emotion regulation to be an automatized strategy whereas others consider it to be implicit disengagement of deliberative process, to date, its neural correlates have been poorly investigated. In addition, the valence specificity of automatic adaptive emotion regulation and levels of activation relative to the neutral condition are controversial; the former is relevant to the attribution of resilient emotion regulation to positivity bias or emotional stability, and the latter to determining whether regulation is based on emotion-specific or emotion-non-specific processes. In this functional magnetic resonance imaging (fMRI) study, we presented positive and negative emotional pictures to healthy young participants and investigated the neural correlates of automatic adaptive emotion regulation in spontaneous emotional response. A significant negative trait effect (i.e., regression coefficient) on activation was identified both for positive and negative emotional responses in various cortical regions. A cluster analysis identified three clusters among these regions based on the valence specificity of the trait effect and level of activation relative to neutral stimuli. Cluster 1 included regions in the sensorimotor cortex characterized by negative emotion-specific decreases in activation relative to neutral stimuli in adaptive individuals. Cluster 2 included several cortical regions including the bilateral dorsal executive network, anterior cingulate, and inferior frontal gyrus, which were characterized by valence-independent decreases in activation in adaptive individuals. Cluster 3 included the bilateral ventrolateral and dorsomedial prefrontal cortices, right insula, and other posterior regions, which were characterized by increased activation for negative stimuli in non-adaptive individuals. These findings support the assumption that automatic adaptive emotion regulation involves the implicit disengagement of deliberative process and suggest the relevance of different cortical networks to the potential emotion- and valence-specificity of adaptive regulation.

## 1. Introduction

Emotionally stressful situations are inevitable in daily life and may be particularly frequent under adverse circumstances such as disease or disaster. Although people may try to cope with such situations by solving the underlying problem (problem-focused coping), if this appears impossible, they must endure the situation by regulating their emotion to reduce the magnitude of its adverse impacts (emotion-focused coping) (Folkman and Lazarus, 1980; Carver et al., 1989; Garnefski et al., 2001). Early research explored the adaptive emotion regulation strategy and typically identified reappraisal of a situation using benign or positive interpretation as the most adaptive, by showing the relationship between the more capacity or frequency of its daily use and the less psychopathology (Gross, 1998; Garnefski et al., 2001; Aldao et al., 2010). The successful coping was typically discussed in the context of explicit or instructed choice and implementation of such adaptive regulation strategies, in part motivated by its application in cognitive behavioral therapy (Smits et al., 2012).

Adaptive emotion regulation also occurs automatically, which explains successful emotion-focused coping in stressful situations in daily life (Bargh and Williams, 2007; Fiori, 2009; Koole et al., 2015b); this type of emotion regulation may be much more common than instructed use or strategic choice. There are two distinct views on the processes underlying automatic adaptive emotion regulation. Some researchers consider it to consist of automatized recruitment of the emotion regulation strategy, i.e., spontaneous and potentially implicit use of the same adaptive emotion regulation strategy (e.g., reappraisal) used in explicit emotional regulation (Bargh and Williams, 2007; Koole et al., 2015b). Such automatic emotion regulation is assumed to develop over frequent use. Other researchers consider the automatic adaptive emotion regulation process to be independent of explicit regulation (Moon and Lord, 2006; Fiori, 2009; Wenzel et al., 2020), in an implicit disengagement of deliberative process that has been shown to occur in the early subliminal phase (Moon and Lord, 2006) and is characterized by acceptance of a situation without judgment or evaluation (Wenzel et al., 2020).

Exploration of the neural correlates of automatic adaptive emotion regulation remains in its infancy. Many studies have investigated the processes underlying explicit adaptive emotion regulation, mainly by examining activation during instructed reappraisal of negative emotional stimuli (Buhle et al., 2014; Frank et al., 2014; Kohn et al., 2014; Morawetz et al., 2017b). Automatic adaptive emotion regulation is associated with activation in wide range of frontoparietal cortices, including the dorsal executive network, emotion processing areas such as the ventrolateral and medial prefrontal cortices, and insula; it also accompanies deactivation of the limbic emotion response system (e.g., the amygdala). A few studies have investigated automatic adaptive emotion regulation by examining individual differences in activation (i.e., related to successful regulation) during the passive presentation of emotional stimuli, i.e., without explicit instruction of emotion regulation or conscious monitoring. One study passively presented pictures of negative facial expressions (e.g., anger or fear) and demonstrated higher and lower neural responses in the prefrontal cortex and amygdala, respectively, in individuals with high reappraisal scores on the Emotion Regulation Questionnaire (ERQ; Gross and John, 2003); this pattern suggested that automatic emotion regulation was driven by automatized strategic regulation (Drabant et al., 2009). Two studies compared neural responses to negative emotional stimuli between controls and experienced meditation practitioners who were considered to have a high automatic emotion regulation capacity. One used noxious heat stimuli and Zen meditators (Grant et al., 2011) and the other used negative emotional pictures and yoga meditators (Froeliger et al., 2012). In both studies, the experienced meditators had lower prefrontal cortex neural responses than control groups, suggesting that automatic emotion regulation consists of implicit disengagement of deliberative processes. However, these studies had reservations about a possible association between individual differences and automatic emotion regulation capacity. The ERQ is a theoretically constructed measure of the capacity for reappraisal, mainly in the context of conscious attempts. Meditation has been documented to affect emotion regulation as well as various physical and psychological measures (e.g., anxiety, depression, life satisfaction; Lomas et al., 2017; Creswell et al., 2019); long-term commitment to meditation has also been associated with pre-existing brain functional differences (Mascaro et al., 2013).

The recently developed Power to Live questionnaire includes an adaptive automatic emotion regulation trait measure (Sugiura et al., 2015), which may be a promising tool for investigating the mechanisms of adaptive automatic emotion regulation. The Power to Live questionnaire measures eight major psychobehavioral characteristics relevant to survival, which were identified through interviews with 1,400 survivors of the 2011 Great East Japan Earthquake and Tsunami disaster, followed by factor analysis (Sugiura et al., 2015). The emotion regulation factor is composed of four items: “During difficult times, I endeavor not to brood,” “During difficult times, I endeavor to think positively, telling myself that this experience will benefit me in the future,” “During difficult times, I compare my circumstances with the situation around me and in society, and I think that matters cannot be helped,” and “When something happens, I try to stay calm and not panic.” These items largely correspond to four of the nine strategies that are considered to be used by people who have experienced negative life events (Cognitive Emotion Regulation Questionnaire, CERQ; Garnefski et al., 2001): specifically, rumination (reversed), positive reappraisal, putting into perspective, and acceptance. The four emotion regulation items were well correlated (Cronbach’s α = 0.77) and the construct was validated through confirmatory factor analyses using data from normative populations (Ishibashi et al., 2019; Matsuzaki et al., 2022). The adaptability of the factor was demonstrated by its positive contribution to survival in various phases of disaster: immediate tsunami evacuation in the initial phase, refugee-related problem solving (Sugiura et al., 2015) and positive perception of public support (Sugiura et al., 2021) in the emergency response phase, and housing recovery and wellbeing in the recovery phase (Sugiura et al., 2015; Sato et al., 2021). This factor has a conceptual advantage as a summary measure of adaptive automatic emotion regulation in that it was empirically and primarily identified as an independent adaptive factor among other adaptive traits, in contrast to the other measures such as ERQ and CERQ scores, which were theoretically constructed as multidimensional models for different emotion regulation strategies and *post hoc* tests of adaptability in each dimension. For example, one CERQ factor (refocus on planning) largely corresponds to the problem solving Power to Live factor.

In exploring the neural correlates of the automatic adaptive emotion regulation using the Power to Live emotion regulation trait measure, two issues are worth further consideration: valence specificity and levels of activation relative to neutral stimuli. Valence specificity is relevant to adaptive emotion regulation in the context of resilience; some researchers have focused on positive appraisal of negative stimuli (i.e., positivity bias) as the key mechanism protecting against the detrimental effects of stress (Kalisch et al., 2015), whereas others have suggested the importance of counter-regulating both negative and positive emotions for psychological adaptation by maintaining a steady emotional balance (Koole et al., 2015a). However, because relevant fMRI studies have examined activation only for negative stimuli (Drabant et al., 2009; Grant et al., 2011; Froeliger et al., 2012), the same trait effect on activation should also be examined for positive stimuli. Previous studies have also assumed a higher level of activation for negative than for neutral stimuli, generally testing how a trait affects increases in activation levels in emotion-specific processes. This assumption largely depends on the process model of emotion (Gross, 1998), in which regulation processes are triggered by an early valuation of emotion and reduce the negative effect of emotional response at a late valuation process. By contrast, some studies appear to suggest that activation levels are lower for negative stimuli than for neutral stimuli (Grant et al., 2011), in which case the basic tenets of emotion regulation must be reconsidered.

In this functional magnetic resonance imaging (fMRI) study, we investigated the neural correlates of automatic adaptive emotion regulation in the spontaneous response to emotional stimuli. We presented positive and negative emotional pictures to healthy young participants and measured their neural responses. We explored the correlation of these responses with adaptive emotion regulation according to the emotion regulation factor of the Power to Live questionnaire (Sugiura et al., 2015). To address the mechanism underlying spontaneous emotion regulation, we avoided providing any explicit instructions or prohibiting emotion regulation or self-evaluation of emotional state. We were interested in whether the effects of adaptive emotion regulation (as reflected in regression coefficients) on activation of the neural correlates of explicit emotion regulation are positive or negative, i.e., whether the frontoparietal cortices would show higher or lower activation in adaptive individuals. Positive and negative effects were predicted under the assumptions of automatized strategic regulation (Bargh and Williams, 2007; Koole et al., 2015b) and implicit disengagement of deliberative processes (Moon and Lord, 2006; Fiori, 2009; Wenzel et al., 2020), respectively. To further characterize the functions of the identified regions, we examined the valence specificity of the trait effect on activation and level of activation relative to that for neutral stimuli. Emotion regulation was predicted to be influenced primarily by negative emotions, under the assumption that positivity bias is adaptive (Kalisch et al., 2015), and to be common for both emotional valances, under the assumption that the maintenance of steady emotional balance is adaptive (Koole et al., 2015a). Previous studies have generally predict higher activation levels for emotional stimuli than for neutral stimuli; however, one study predicted the opposite response (Grant et al., 2011). We also performed a cluster analysis of the identified regions based on valence specificity of the trait effect and activation levels relative to neutral stimuli to identify functional networks comprising subsets of regions with similar functional characteristics. This approach complements the separate tests for each characteristic in each region by allowing the identification of networks that integrate both characteristics without spurious dichotomization using a specific statistical threshold (Chen, 2022).

## 2. Materials and methods

### 2.1. Participants

We enrolled 47 healthy, right-handed, young adult students *via* advertisements placed around Tohoku University. All participants were native Japanese speakers and had no history of neurological or psychiatric illnesses. Data from 40 participants (19 females, 21 males; mean age ± standard deviation [SD] = 21.9 ± 1.8) were analyzed. Seven participants were excluded because of deficient MRI data (*n* = 5) or excessive head movement during MRI (>3 mm; *n* = 2). The study was approved by the Institutional Review Board of the Graduate School of Medicine of Tohoku University, Japan, and was conducted in accordance with the Declaration of Helsinki. Written informed consent was obtained from all participants.

### 2.2. Trait measure of adaptive automatic emotion regulation

As a trait measure of adaptive automatic emotion regulation, we used the emotion regulation factor of the Power to Live questionnaire (Sugiura et al., 2015), which measures eight survival-related psychological and behavioral factors: leadership, problem solving, altruism, stubbornness, etiquette, self-transcendence, active wellbeing, and emotion regulation. The questionnaire includes 34 items related to ways of thinking, daily attitudes, and habits; some are related to stressful situations in general, whereas others appear to be unrelated. Respondents rate the self-applicability of each item on a six-point scale (0: not at all; 5: very much). Each factor consists of three to five items; item scores are averaged and converted to% maximum values to generate a factor score. Although we focused on emotion regulation in this study, the mean score of the eight factors was calculated as a covariate or to standardize emotion regulation scores to adjust for general tendencies in trait questionnaire responses among participants; i.e., some participants show an overall preference for high or low scores, irrespective of the question.

### 2.3. Experimental tasks

**FIGURE 1:**
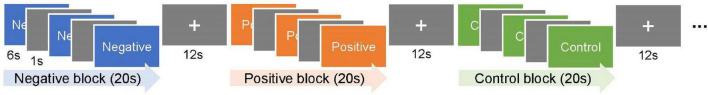
Experimental design. Each block consisted of a serial presentation of three emotional pictures with the same valence, i.e., negative, positive, or neutral (control), from the International Affective Picture System. The triad of three blocks with different valences, with the order counterbalanced across participants, was repeated six times. The participants pressed the button after they had sufficiently appreciated each picture to ensure their wakefulness.

We presented each participant with 18 negative (codes: 2691, 3500, 6213, 6260, 6300, 6312, 6821, 6834, 6838, 8485, 9007, 9280, 9342, 9424, 9471, 9830, 9910, and 9925), 18 positive (codes: 1440, 1441, 1710, 2091, 2260, 2311, 2331, 2332, 2340, 2345, 2387, 2530, 4614, 4626, 5201, 5833, 7325, and 8185), and 18 neutral (i.e., control) (codes: 2840, 2980, 5390, 5471, 5534, 7000, 7004, 7009, 7020, 7050, 7052, 7130, 7150, 7161, 7187, 7211, 7705, and 7950) pictures from the International Affective Picture System (IAPS) (Lang et al., 2008). Each session had a block design (Figure 1) with each 20-s block composed of showing three pictures with the same valence category (each 6-s presentation separated by a 1-s eye-fixated rest). The triad of three blocks with different valences, with their order counterbalanced across participants, was repeated six times with a 12-s eye-fixated rest being inserted between consecutive blocks and at the beginning and end of the session, resulting in a total session duration of 588 s.

Each participant was placed in a supine position on the MRI scanner bed; the head was immobilized using a band and elastic blocks. Visual stimuli were presented on a translucent screen from an LCD projector. The participants viewed the stimuli using a mirror attached to the head coil. They were instructed to press the button of a fiber optic response pad (Current Designs Inc., Philadelphia, PA, USA) with their right index finger after they had sufficiently appreciated each picture to ensure their wakefulness. Stimulus presentation and response collection were controlled using the Presentation software (Neurobehavioral Systems, Inc., Berkeley, CA, USA).

### 2.4. fMRI data acquisition and pre-processing

The fMRI time-series dataset of whole-brain T2*-weighted gradient echo-echo planar imaging (EPI) scans was acquired using a 3-Tesla Philips Achieva scanner (Philips Medical Systems, Best, Netherlands). The entire cerebrum was covered in 33 transaxial images (echo time = 30 ms, flip angle = 70°, slice thickness = 3.0 mm, slice gap = 0.0 mm, field of view = 192 mm, matrix = 64 × 64, voxel size = 3 mm × 3 mm × 3 mm) at a repetition time of 2,000 ms. The time-series dataset for each participant consisted of 294 scans obtained during the 588-s session.

Statistical Parametric Mapping (SPM12; Wellcome Department of Imaging Neuroscience, London, UK) was used for data preprocessing and analyses. The preprocessing procedure included correction for head motion, adjustment of acquisition timing across slices, spatial normalization using an EPI template of the Montreal Neurological Institute (MNI), and smoothing using an isotropic Gaussian kernel with an 8-mm full-width at half-maximum.

### 2.5. fMRI data analysis

A conventional two-level approach for a multi-subject time-series dataset was adopted. At the first (within-subject) level, the condition-specific neural activation of each participant was estimated using a voxel-by-voxel multiple regression analysis of the time course. A general linear model including a regressor for each valence (i.e., negative, positive, and control) was constructed by assuming a 20 s neural activation during each block and using the conventional hemodynamic response function. Six head-motion parameters (three for translation and three for rotation) estimated during the head-motion-correction process were included as covariates. A high-pass filter with a cutoff frequency of 1/128 Hz was applied for detrending. Activation images for negative and positive emotional responses were generated using the contrast estimate (beta) of negative–control and positive–control, respectively.

At the second (between subjects) level, the neural correlates of the automatic adaptive emotion regulation trait were explored using separate voxel-by-voxel multiple regression analyses of activation images for negative and positive emotional responses. The emotion regulation score was included as an independent variable of interest (i.e., a measure of adaptive automatic emotion regulation trait). Age, sex, and the mean Power to Live score (i.e., to adjust for general tendencies in trait questionnaire responses) were included as covariates. Both positive and negative effects of emotion regulation were explored in terms of regression coefficients of the trait score. Activation clusters of significant effects were initially identified using a threshold of *p* < 0.001 (uncorrected) and then at a cluster-level extent threshold (*p* < 0.05) based on random field theory was applied to control for family wise error, assuming the entire cerebrum as the search volume (Friston et al., 1994).

To further characterize the functions of the identified regions, we examined the valence specificity of trait effects and activation levels relative to neutral stimuli at each peak voxel. To examine valence specificity, the trait effect was also tested for the opposite valence, and the results were compared. To examine activation levels relative to neutral stimuli, we performed separate one-sample *t*-tests on the average activation for negative and positive emotional responses (i.e., negative vs. control and positive vs. control, respectively) across participants. The threshold for statistical significance was *p* < 0.05, uncorrected for multiple comparisons (because we were interested in two characteristics of the activation pattern at each voxel).

To identify regional subsets with similar functional characteristics (i.e., valence specificity and activation levels relative to neutral stimuli), we applied a hierarchical cluster analysis to all identified regions in the four voxel-wise analyses (i.e., positive and negative trait effects on activation for negative and positive emotional responses). As a dissimilarity measure across regions, we determined Euclidian distances using the set of four *t*-values (i.e., trait effect and average activation for emotional responses in two valences) at each peak voxel. Ward’s method, which minimizes the total within-cluster variance, was used for clustering (Ferreira and Hitchcock, 2009). Principal component analysis was applied to the same dataset to visualize the distribution of regions.

## 3. Results

### 3.1. Emotion regulation score

The emotion regulation factor scores (% maximum; mean ± SD) were 60.1 ± 18.5 and Cronbach’s α was 0.74 for the analyzed participants (*n* = 40).

### 3.2. Neural correlates of the automatic adaptive emotion regulation trait

**FIGURE 2:**
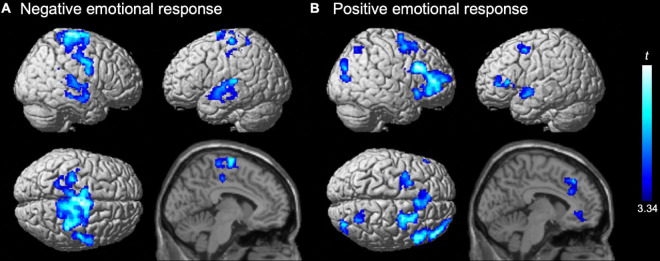
Negative effects of the emotion regulation trait on activation for **(A)** negative and **(B)** positive emotional responses. Significant effects (*t*-values) of the emotion regulation score (power to live questionnaire) in the second-level multiple regression model are represented by a blue-cyan scale, rendered on the surface and overlaid on the parasagittal section (*x* = –6) of an SPM12 standard structural brain image. Statistical significance was determined at a cluster-forming threshold of uncorrected *p* < 0.001 and then corrected to *p* < 0.05 (family wise error) for the cluster size. No positive trait effect was detected.

On activation for the negative emotional response (i.e., negative vs. control), a significant positive effect of the emotion regulation score was not detected. A significant negative effect of the score was detected in the bilateral sensorimotor cortex, including the central sulcus, pre-central and paracentral gyri, and supplementary motor area, as well as the bilateral temporal cortices, including multiple regions in the superior and middle temporal gyri along the posterior–anterior axes (Table 1 and Figure 2A). The finding supports the assumption that automatic adaptive emotion regulation involves the implicit disengagement of deliberative processes (Moon and Lord, 2006; Fiori, 2009; Wenzel et al., 2020).

**TABLE 1:** Neural correlates of the automatic adaptive emotion regulation trait for negative emotional response.

Structure		Coordinate			Trait effect			Average	Positive–Control		Trait	Cluster	
		x	y	z	*t*	k	*p*	*t*	Trait	Average	N–P	/Label	
Central sulcus	L	−30	−24	64	−5.15a			−2.92*	−2.26*	1.14	−1.84*	1	LCS
	R	34	−30	64	−5.61a	5134	<0.001	−4.02*	−3.01*	−0.24	−1.85*	1	RCS
Pre-central gyrus	R	22	−20	72	−5.55a			−3.39*	−2.50*	0.88	−2.73*	1	RPreCG
	R	52	−2	34	−4.83	734	0.001	−2.63*	−1.88*	−0.37	−2.13*	1	RPreCS
Paracentral gyrus (posterior)	L	−8	−28	66	−4.39a			−2.07*	−0.43	1.36	−2.38*	1	LpParaCG
Paracentral gyrus (anterior)	R	8	−18	74	−5.11a			−1.93*	−2.24*	0.52	−2.84*	1	RaParaCG
Paracentral gyrus (posterior)	R	12	−28	60	−4.77a			−2.55*	−1.62	0.39	−1.64	1	RpParaCG
Supplementary motor area	L	−6	−10	74	−4.91a			−2.16*	−0.96	−0.68	−2.57*	1	LSMA
	R	8	−4	62	−5.46a			−3.27*	−2.10*	−0.99	−2.04*	1	RSMA
Superior temporal gyrus (posterior)	L	−38	−32	14	−4.91c	979	<0.001	−3.57*	−3.21*	−2.56*	−0.47	2	LpSTG
	R	40	−36	10	−5.48b	1041	< 0.001	−5.84*	−2.19*	−2.39*	−1.65	2	RpSTG
Superior temporal gyrus (middle)	L	−62	−26	6	−3.97c			−3.91*	−2.75*	−3.75*	−1.57	2	LmSTG
	R	62	−18	2	−4.38b			−6.37*	−2.35*	−3.56*	−0.82	2	RmSTG
Superior temporal gyrus (anterior)	L	−56	−4	−8	−4.76c			−2.19*	3.41*	−1.69*	−0.65	2	LaSTGn
	R	54	−6	−10	−3.91b			−2.26*	−2.94*	−1.41	−0.77	2	RaSTG
Middle temporal gyrus (middle)	R	56	−30	−12	−4.34b			2.5*	−2.71*	0.88	−1.13	3	RmMTG
Middle temporal gyrus (anterior)	R	62	−10	−18	−4.59b			4.48*	−1.93*	1.19	−1.23	3	RaMTG

**FIGURE 3:**
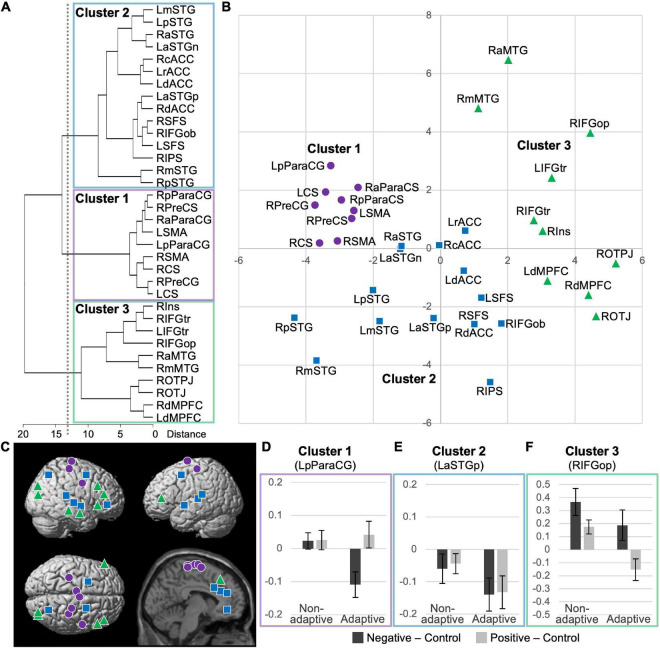
Results of cluster analysis of 34 identified regions with significant trait effects based on *t*-values for trait effect and average activation for emotional responses in two valences. **(A)** Dendrogram of the hierarchical cluster analysis; a threshold of 13.96 for between-cluster distances (dotted line) resulted in a three-cluster solution. **(B)** Two-dimensional plots of principal components. See Tables 1, 2 for anatomical labels. **(C)** Anatomical locations of the peaks, indicated by cluster-specific symbols. **(D–F)** Activation profiles of the representative regions of clusters 1–3 (i.e., the posterior left paracentral gyrus, the anterior left superior temporal gyrus, and opercular part of the right inferior frontal gyrus, respectively). Average emotional responses for two valences are shown separately for non-adaptive (*n* = 20) and adaptive (*n* = 20) groups, based on a median-split of low and high emotion regulation scores, respectively.

For each peak voxel, laterality (L, left; R, right), MNI coordinate, *t*-value for the trait effect, and the associated cluster are given. The number of voxels (k) and *p*-value are given at the highest peak voxel. Letters that are the same indicate belonging to the same cluster. To further characterize each peak voxel, the *t*-value for average activation (i.e., one-sample *t*-test) for the negative emotional response, for the trait effect and average activation for the positive emotional response, and for a difference in the trait effect between the negative and positive emotional responses (negative–positive) are given (*p < 0.05, uncorrected). The rightmost column shows the cluster number and abbreviation for anatomical name used in Figure 3.

We also did not detect a significant positive effect of the emotion regulation score on activation for the positive emotional response (i.e., positive vs. control). A significant negative effect of the score was identified in multiple prefrontal regions, including the bilateral superior frontal sulci (dorsolateral prefrontal cortex; dlPFC), inferior frontal gyri (ventrolateral prefrontal cortex; vlPFC), and dorsomedial prefrontal cortex (dmPFC). Furthermore, significant negative effects were observed in several regions in the bilateral anterior cingulate cortex (ACC), right insula, intraparietal gyrus, occipito-temporal and occipito-temporo-parietal junctions, and the left superior temporal gyrus (Table 2 and Figure 2B). This result also supports the assumption of implicit disengagement of deliberative processes.

**TABLE 2:** Neural correlates of the automatic adaptive emotion regulation trait for positive emotional response.

Structure		Coordinate			Trait effect			Average	Negative–Control		Trait	Cluster	
		x	y	z	*t*	k	*p*	*t*	Trait	Average	N–P	/Label	
Inferior frontal gyrus (orbitalis)	R	34	36	−4	−5.49a			−4.08*	−2.15*	−1.95*	1.19	2	RIFGob
Superior frontal sulcus	L	−24	6	50	−5.45	436	0.003	−3.51*	−3.49*	−1.65	0.4	2	LSFS
	R	22	14	50	−5.05b	1719	0	−3.88*	−2.54*	−2.48*	0.69	2	RSFS
Anterior cingulate cortex (rostral)	L	−14	40	2	−4.94	291	0.021	−0.73	−2.63*	−1.11	0.49	2	LrACC
Anterior cingulate cortex (dorsal)	L	−8	28	28	−4.24b			−1.97*	−1.94*	−1.87*	0.82	2	LdACC
	R	8	40	24	−3.93b			−4.03*	−1.78*	−2.44*	0.55	2	RdACC
Anterior cingulate cortex (caudal)	R	8	16	36	−4.32b			−1.41	−3.59*	−1.63	0.03	2	RcACC
Intraparietal sulcus	R	28	−62	50	−5.19	266	0.029	−5.86*	−2.12*	−3.20*	1.15	2	RIPS
Superior temporal gyrus (anterior)	L	−50	−6	−8	−4.23	234	0.045	−3.47*	−3.18*	−3.14*	0.17	2	LaSTGp
Inferior frontal gyrus (opercularis)	R	50	20	30	−6.37a	2366	0	0.27	−2.45*	3.7*	0.78	3	RIFGop
Inferior frontal gyrus (triangularis)	L	−50	30	6	−4.23	282	0.023	−1.91*	−3.48*	2.74*	0.73	3	LIFGtr
	R	40	32	20	−5.17a			−1.69*	−2.23*	0.84	0.79	3	RIFGtr
Insula	R	44	20	−10	−4.28a			−2.71*	−2.24*	1.16	0.61	3	Rins
Dorsomedial prefrontal cortex	L	−6	32	44	−4.57b			−3.66*	−1.04	−0.01	1.65	3	LdMPFC
	R	6	28	48	−5.03b			−4.68*	−0.69	0.69	1.84*	3	RdMPFC
Occipitotemporal junction	R	38	−82	14	−4.57c	312	0.016	−6.54*	−2.07*	1.12	1.65	3	ROTJ
Occipito temporo parietal junction	R	34	−82	30	−4.11c			−5.12*	−1.32	2.45*	1.65	3	ROTPJ

To further characterize each peak voxel, *t*-values for the trait effect and average activation for the negative emotional response are given. Other details are the same as for Table 1.

### 3.3. Functional characterization of the identified regions

For regions that showed a significant trait effect on activation for the negative emotional response, functional characteristics including valence specificity of the trait effect and activation levels relative to neutral stimuli are summarized in Table 1. Regarding the valence specificity of the trait effect, the negative trait effect was significantly larger for negative than positive emotional responses in most identified regions of the bilateral sensorimotor cortex, supporting the assumption that positivity bias is adaptive (Kalisch et al., 2015). By contrast, in all identified regions of the bilateral temporal cortices, the negative trait effect was significant for both negative and positive emotional responses, with no significant between-valence difference, supporting the assumption that maintenance of steady emotional balance is adaptive (Koole et al., 2015a). Average activation was significantly lower for negative stimuli than for neutral stimuli in all identified regions except the bilateral middle temporal gyri, and the pattern was similar in the average activation for positive stimuli in the identified temporal cortical regions. This result contradicts current views about the regulation of activation for emotional response.

The functional characteristics of regions that showed a significant trait effect on activation for positive emotional response are summarized in Table 2. Regarding the valence specificity of the trait effect, the negative trait effect was significant for both positive and negative emotional responses, with no significant between-valence differences in most regions, supporting the assumption that maintenance of steady emotional balance is adaptive. In the bilateral dmPFC and right occipito-temporo-parietal junction, the trait effect was not significant for negative emotional response, and the trait effect for positive emotional response was significantly larger than for negative emotional response in the right dmPFC. Average activation was significantly lower for positive stimuli than for neutral stimuli in most identified regions, and the pattern was similar in the average activation for negative stimuli, except in the right occipito-temporo-parietal junction.

### 3.4. Cluster analysis

The results of hierarchical cluster analysis for all 34 identified regions (i.e., pooled data for identified regions with a trait effect for emotional response in both valences) based on their functional characteristics (i.e., *t*-values for trait effect and average activation for emotional response in both valences) are shown as a dendrogram in Figure 3A. The degrees of similarity in functional characteristics among regions were visualized as two-dimensional plots of the loadings for the first and second principal components, which explained 41 and 36% of the total variance, respectively (Figure 3B). We chose a three-cluster solution based on a threshold of 13.96 for between-cluster distances, where cluster 1 was dissociated from cluster 2 (Figure 3A). Further lowering the threshold divided cluster 3, which seemed inappropriate based on the visual inspection of its distribution (Figure 3B). The anatomical distribution of the regions (i.e., peaks) of each cluster is represented by symbols on the brain surface and section in Figure 3C. To visualize the functional characteristics of each cluster, the average activation for emotional responses in the two valences are shown separately for adaptive (*n* = 20) and non-adaptive (*n* = 20) groups in the representative region (Figures 3D–F). The groups were determined based on the median emotion regulation score (standardized using the mean Power to Live score), which resulted in average ± SD scores of 1.12 ± 0.17 and 0.77 ± 0.15, respectively. There were no significant differences between groups in age (22.0 ± 1.9 and 21.9 ± 1.7 years, respectively; *p* = 0.930) or sex (male/female: 12/8 and 9/11, respectively; χ*^2^* = 0.902, df = 1, *p* = 0.342).

Cluster 1 included all nine regions identified in the sensorimotor cortex. In these regions, the trait effect was largely specific to negative emotional response and average activation was significantly lower for negative stimuli than for neutral stimuli (Table 1). These two characteristics appear to correspond with a negative emotion-specific decrease in activation relative to neutral stimuli in adaptive individuals (Figure 3D).

Cluster 2 was composed of the dorsal executive network (i.e., dlPFC and intraparietal sulcus), multiple regions in the bilateral ACC and superior temporal gyri, and the orbital part of the right inferior frontal gyrus. In these regions, the trait effect was largely significant for both negative and positive valences, with no significant between-valence difference, and average activation was significantly lower for both emotional stimuli than for neutral stimuli (Tables 1, 2). These two characteristics appear to correspond with a valence-independent decrease in activation relative to neutral stimuli in adaptive individuals (Figure 3E).

Cluster 3 was composed of the bilateral vlPFC and dmPFC, right insula, middle temporal gyrus, and posterior occipitoparietal regions. In these regions, the trait effect was largely significant for both negative and positive valences, with no significant between-valence difference, and average activation tended to be higher for negative stimuli and lower for positive stimuli than for neutral stimuli (Tables 1, 2). These two characteristics appear to correspond to a complicated activation pattern in the opercular part of the right inferior frontal gyrus. Compared to that for neutral stimuli, activation for negative stimuli showed a greater increase in non-adaptive than in adaptive individuals, whereas that for positive stimuli appeared to increase in non-adaptive and decrease in adaptive individuals (Figure 3F). This pattern is consistent with the regulation of activation for emotional response.

## 4. Discussion

We explored the neural correlates of automatic adaptive emotion regulation in spontaneous responses to emotional stimuli; we also characterized the functions of these regions in terms of the valence specificity of the trait effect on activation and activation levels relative to neutral stimuli. A significant positive effect of the trait score on activation was not detected for either negative or positive emotional response. A significant negative effect of the score on activation was identified in the bilateral sensorimotor and temporal cortices for negative emotional response and in the bilateral prefrontal cortices, ACC, right insula, intraparietal, and other posterior areas for positive emotional response. This finding supports the assumption that automatic adaptive emotion regulation is the implicit disengagement of deliberative processes (Moon and Lord, 2006; Fiori, 2009; Wenzel et al., 2020). Regarding the valence specificity of the trait effect, the effect was predominant for negative emotional response in the bilateral sensorimotor cortex, supporting the assumption that positivity bias is adaptive (Kalisch et al., 2015), whereas the effect was equally significant for negative and positive emotional responses in other regions, supporting the assumption that maintenance of steady emotional balance is adaptive (Koole et al., 2015a). Significantly higher average activation levels for emotional stimuli, which are predicted by the regulation of activation for emotional response, were identified only in a few regions, such as the bilateral middle temporal gyri and the right occipito-temporo-parietal junction. In most other regions, average activation was significantly lower for emotional stimuli than for neutral stimuli. The cluster analysis results suggested three clusters among the identified regions. Cluster 1 included regions in the sensorimotor cortex characterized by negative emotion-specific decreases in activation relative to neutral stimuli in adaptive individuals. Cluster 2 included several cortical regions including the bilateral dorsal executive network, ACC, superior temporal gyri, and the orbital part of the right inferior frontal gyrus, which are characterized by valence-independent decreases in activation in adaptive individuals. Cluster 3 included the bilateral vlPFC and dmPFC, right insula, middle temporal gyrus, and posterior occipitoparietal regions, which are characterized by a complicated activation pattern. For negative stimuli, activation increases relative to neutral stimuli were higher in non-adaptive than in adaptive individuals, consistent with the regulation of activation for emotional response; however, for positive stimuli, activation increased in non-adaptive and decreased in adaptive individuals.

Although recent studies have supported implicit disengagement of deliberative processes as the mechanism underlying automatic adaptive emotion regulation (Moon and Lord, 2006; Fiori, 2009; Wenzel et al., 2020), previous neural evidence obtained from studying experts in Zen (Grant et al., 2011) and yoga meditators (Froeliger et al., 2012) have had methodological issues in that meditation affects not only emotion regulation but also various physical and psychological measures (Lomas et al., 2017; Creswell et al., 2019) and is associated with preexisting functional differences in the brain (Mascaro et al., 2013). In this study, we overcame this limitation by enrolling healthy individuals and adopting a parametric approach using a trait measure for automatic adaptive emotion regulation, and obtained more robust evidence for the predominantly negative effect of automatic adaptive emotion regulation on activation of emotional responses. The meditation studies by Grant et al. (2011) and Froeliger et al. (2012) enrolled relatively few participants (13 and 14, respectively), whereas we enrolled a larger sample (*n* = 40), which may also have contributed to our ability to identify trait effects in more extensive regions than these previous studies.

In addition to the role of the frontoparietal cortices, the involvement of the amygdala is another difference between the two potential mechanisms of automatic adaptive emotion regulation. Suppression of the amygdala response by prefrontal functioning is a key feature of explicit reappraisal (Buhle et al., 2014; Frank et al., 2014; Kohn et al., 2014). Previous studies of individual differences have reported mixed results. One study demonstrated association of higher reappraisal scores in ERQ and lower neural responses in the amygdala (Drabant et al., 2009) and another study on Zen meditators reported greater amygdala deactivation in experts than in controls (Grant et al., 2011). On the other hand, the study on yoga meditators showed an absence of the dlPFC–amygdala correlation (Froeliger et al., 2012). In line with the latter, we did not detect a trait effect in the amygdala, despite our liberal statistical threshold (uncorrected *p* < 0.05), which supports the implicit disengagement of deliberative processes.

Although acceptance appears to be conceptually closer to automatic adaptive emotion regulation, our findings suggest that these are at least in part distinct concepts. Acceptance of the reality of a stressor together with an absence of an active coping strategy is an adaptive emotion regulation strategy distinct from reappraisal (Aldao et al., 2010). Some researchers regard it as a subtype of reappraisal strategy (McRae et al., 2012a), while others consider it an essential aspect of mindfulness and related therapy (Bishop et al., 2004; Hayes et al., 2006) and thus a form of automatic adaptive emotion regulation (Wenzel et al., 2020). Although a limited number of studies have examined the neural basis of acceptance, a recent meta-analysis identified decreased brain activity in the posterior cingulate cortex or precuneus as a common finding (Messina et al., 2021). Although the association of regulation with decreased activation is consistent with the implicit disengagement of deliberative processes (Moon and Lord, 2006; Fiori, 2009; Wenzel et al., 2020), the identified regions did not overlap with our current finding on the automatic adaptive emotion regulation trait.

The current finding on the valence specificity of the trait effect appears to reconcile the discussion on the nature of adaptive emotion regulation in the context of resilience; that is, different cortical networks showed activation patterns supporting distinct mechanisms. Regions in the sensorimotor cortex (cluster 1) showed negative emotion-specific trait effects, supporting the assumption of positivity bias adaptiveness, where positive appraisal of negative stimuli (i.e., positivity bias) is a key mechanism protecting against the detrimental effects of stress (Kalisch et al., 2015). Deactivation or suppression of the sensorimotor cortex has not been detected in meta-analyses of reappraisal (Buhle et al., 2014; Frank et al., 2014; Kohn et al., 2014; Morawetz et al., 2017b) or acceptance (Messina et al., 2021). However, it has been reported in some studies as reduced activation during explicit reappraisal compared to the natural viewing of negative pictures (McRae et al., 2012b; Morawetz et al., 2017a). In patients with borderline personality disorder, which is characterized by poor emotion regulation, increased activation of the sensorimotor cortex is observed for negative but not positive images (Koenigsberg et al., 2009). It may be possible to link deactivation of the sensorimotor cortex with suppression of the emotional response in the physiological domain, given the association between sensorimotor activation and physiological emotional markers during the viewing of negative (vs. positive) images (Anders et al., 2004). However, the remaining regions identified as having a trait effect in this study (clusters 2 and 3) showed valence-independent trait effects for emotional responses, supporting the assumption of adaptiveness in the maintenance of steady emotional balance by counter-regulating both negative and positive emotions (Koole et al., 2015a).

Our findings on activation levels relative to neutral stimuli also support and contradict parts of the proposed mechanisms for the regulation of emotion-specific processes. An activation increase for negative stimuli relative to neutral stimuli, with a higher degree in non-adaptive individuals, was observed in regions of cluster 3. In the framework of the classical process model of emotion (Gross, 1998), such a trait effect on activation may reflect inefficient top-down regulation triggered by the early valuation of emotion or unsuppressed emotional responses in the late valuation with adverse psychological impacts. The involvement of various saliency detection systems, such as the insula (Menon and Uddin, 2010) and vlPFC (Vossel et al., 2014), and regions implicated in the elaborative process for socioemotional conflict, such as the middle temporal gyrus, dmPFC, and posterior occipitoparietal region (Wakusawa et al., 2009; Sekiguchi et al., 2013; Oba et al., 2020), in this cluster appears to be consistent with this interpretation. However, a contradictory finding to mechanisms based on the conventional process model was identified in regions of clusters 1 and 2. Significantly lower average activation for emotional stimuli than for neutral stimuli, with a larger degree in adaptive individuals, identified in these regions may suggest the involvement of emotion-non-specific processes as a target of regulation. Thus, adaptive regulation may involve the suppression of emotion-non-specific processes for task execution (e.g., general picture appreciation). The involvement of top-down attention or executive systems including the dlPFC, ACC, intraparietal sulcus (Banich, 2009; Vossel et al., 2014), and regions sensitive to semantic processing loads including the superior temporal gyrus and orbital part of the inferior frontal gyrus (Peyrin et al., 2010; Davis et al., 2011) in cluster 2 is consistent with this idea.

The current findings have important basic and clinical implications regarding the role of the prefrontal control system in emotion regulation. The identified association between high prefrontal activation and low automatic adaptive emotional regulation is congruent with the view that prefrontal activation can index emotional reaction, as demonstrated by the causal effect of visceral stimulation on prefrontal activation (Hamaguchi et al., 2004), and with the general tendency of high prefrontal activation in response to negative emotional stimuli in patients with poor emotion regulation, such as those with borderline personality disorder (Koenigsberg et al., 2009), anorexia nervosa (Seidel et al., 2018), and attention-deficit/hyperactivity disorder (Materna et al., 2019). However, we do not intend to generalize this simple relationship between lower prefrontal activation and better emotional regulation across all contexts or situations. During conscious emotion regulation, reduced prefrontal activation is associated with poor emotion regulation (Picó-Pérez et al., 2017; Wang et al., 2018). Both excitatory and suppressive effects of the prefrontal cortex on the visceral response to emotional stimuli have been demonstrated in recent brain stimulation studies (e.g., Aizawa et al., 2021). The prefrontal control and emotion systems have bidirectional relationships that vary contextually.

In summary, we report a predominantly negative effect of automatic adaptive emotion regulation on activation for emotional responses, supporting a mechanism of implicit disengagement of deliberative processes, rather than automatized strategic regulation. These regions were divided into three subsets/clusters based on functional characterization of the valence specificity of the trait effect on activation and activation levels relative to neutral stimuli. Cluster 1 included regions in the sensorimotor cortex characterized by negative emotion-specific decreases in activation relative to neutral stimuli in adaptive individuals. Cluster 2 included several cortical regions including the bilateral dorsal executive network, ACC, superior temporal gyri, and the orbital part of the right inferior frontal gyrus, which were characterized by valence-independent decreases in activation in adaptive individuals. Cluster 3 included the bilateral vlPFC and dmPFC, right insula, middle temporal gyrus, and posterior occipitoparietal regions, which were characterized by activation increases for negative relative to neutral stimuli in non-adaptive individuals. The identified negative emotion-specific trait effect in cluster 1 and valence-independent trait effect in clusters 2 and 3 support different mechanisms for adaptive emotion regulation in the context of resilience; i.e., adaptiveness in positivity bias and maintenance of steady emotional balance, respectively. The higher activation levels in cluster 3 and lower levels in clusters 1 and 2 relative to neutral stimuli also had different functional implications, with the former supporting existing theories based on the regulation of emotion-specific processes and the latter suggesting the involvement of emotion-non-specific general processes for task execution as a target of regulation. These findings have important basic and clinical implications in understanding of the functional organization of automatic adaptive emotion regulation, which appear to be underpinned by at least three distinct functional networks.

## Data availability statement

The raw data supporting the conclusions of this article will be made available by the authors, without undue reservation.

## Ethics statement

The studies involving human participants were reviewed and approved by the Institutional Review Board of the Tohoku University Graduate School of Medicine, Sendai, Japan. The patients/participants provided their written informed consent to participate in this study.

## Author contributions

MSu, YK, RK, and SF contributed to the study conception and design. YK, TM, MSh, SH, KN, and DT prepared the experimental stimuli and conducted the MRI experiment. MSu performed the statistical analysis and wrote the first draft of the manuscript. All authors contributed to manuscript revision, read, and approved the submitted version.
